# The Eighth Periodical International Ophthalmological Congress

**Published:** 1894-12

**Authors:** Alvin A. Hubbell

**Affiliations:** Buffalo, N. Y.


### Report of Societies.

### THE EIGHTH PERIODICAL INTERNATIONAL OPHTHALMOLOGICAL CONGRESS, HELD AT EDINBURGH, AUGUST 7-11, 1894.

By ALVIN A. HUBBELL, M. D., Buffalo, N. Y.

### HISTORICAL INTRODUCTION.

Ophthalmology was the earliest to become distinctively a special department of medicine, being at first degraded by charlatanism and quackery, but afterwards elevated to its proper position by scientific study and practice; was first to put forth its separate treatises; was first to organize special hospitals; was first to publish special periodical literature; was first to occupy special chairs in medical schools, and was first to establish an international congress.

In 1838, Florent Cunier, of Brussels, Belgium, founded the *Annales d'Oculistique*, the first exclusively ophthalmological journal ever published, and through it an interest was awakened and an influence extended which did more than all else combined to secure for ophthalmology scientific treatment and professional recognition and respect. The maintenance of this journal was an arduous task, but it was successful. In 1853, it was transferred to other hands, in consequence of the early death, in April of that year, of its learned founder and editor. Those who then assumed management were Dr. Louis S. Fallot, of Brussels; Dr. J. Bosch, of Gand; Dr. F. Hairion, of Louvaine; Dr. J. VanRoosbroeck, of Gand, and Dr. E. Warlomont, of Brussels, the latter being made the editor-in-chief. It was this editorial committee, which, following the example of the lamented Cunier in the consecration of its efforts to the scientific advancement of ophthalmology, conceived the idea of calling together from all parts of the civilized world as many as possible who were exclusively, or otherwise closely, identified with this branch of medicine. Without doubt, Dr. Warlomont was the moving spirit in this project, but the whole editorial staff constituted itself a committee of organization, with Dr. Fallot, president, and Dr. Warlomont, secretary-general. In a note to the readers of the *Annales d'Oculistique*, January, 1857, the editorial committee made the following statement:

The study of ophthalmology continues to extend; some men of most eminent merit are consecrating to it their time and labors. That which we have formerly predicted, and that which we have invoked with all our heart, is accomplished today. Besides, ophthalmology has progressed, and it has become specialized. Look at Germany, at England, where it is cultivated with so much ardor and success, and see physicians of greatest renown devoting themselves exclusively to it. We know, and have many times repeated, that from a scientific point of view, ophthalmology cannot be isolated from other branches of the healing art; it receives from them and furnishes to them by a just reciprocity most important information. It is not so in regard to the technical part; here it has its proper domain, its means of diagnosis, its operative manipulations, its instruments and apparatus, and it is not too much for any one life to explore, to search into and to make productive.

It is under the domination of this thought that the editorial committee of these *Annales* has conceived the project of calling together, at Brussels, in the month of September next, in a special congress, men who cultivate ophthalmology, and those who, without specially cultivating it, are interested in it and understand its importance.

This committee sent out its first circular, dated January 15, 1857. Pursuant to this call and announcement, ophthalmologists and physicians interested in ophthalmology to the number of 250 convened at Brussels, September 13-16, 1857, and the "Periodic International Ophthalmological Congress" was inaugurated.

To detail the program that was carried out, to describe the enthusiasm that was aroused in the discussions of the assigned questions, and to note the interest manifested in the ophthalmoscope which had so recently (1851) been discovered by Helmholtz and had so wonderfully opened up new fields of exploration, would be foreign to the purposes of this communication. But something of the *personnel* of this first international ophthalmological congress, and, indeed, so far as I can learn, the first international congress of medicine or any of its branches, may be *apropos*.

While Dr. E. Warlomont was undoubtedly the "father" of the congress, Dr. Fallot was made its president. Dr. Warlomont was elected secretary-general, and as in its preliminary organization, so here he was the most energetic and the most efficient of any in its management. Dr. Fallot was an older man, not exclusively an ophthalmologist, but a most distinguished physician of Belgium, and was worthy of the highest honors of the congress, particularly in view of his coöperation in its organization. Warlomont was a young man, and by his ability and zeal was eminently fitted to perform the duties incumbent upon this office. The success of any society, association or congress depends largely upon its secretary. If he has the proper ability and energy, it will be a success always. Warlomont was the right man here, and this congress was a success, even under much opposition and reproach, and it greatly helped to win for ophthalmology, as a specialty, an honorable recognition that has continued to increase to the present time. Thirty countries, or governments, were represented. Among the members, from our own country, were Dr. S. D. Gross, Dr. Isaac Hays, Dr. Lazarus and Dr. Little, all of Philadelphia; from Great Britain, among others, were Bowman, Dixon, Streatfield, Critchett, Mackenzie and Walton; from Austria, Arlt, Ed. Jæger, Stellwag; from Denmark, Hansen (now Hansen-Grut), Thüne; from Hanover, Max Langenbeck, the two Stromeyers; from Holland, Bauduin, Donders; from Italy, Quaglino, Gioppi, Quadri; from France, Cusco, Desmarres, Guélpin, Sichel, Stœber; from Germany, Juengken, Langenbeck, Von Graefe, etc., etc. These distinguished men and many more honored this first congress by their presence, and enriched its transactions by their communications. But where are they today? Scarcely any live to tell the story of that time. Most of them have gone to their reward, and "their works do follow them," and in many instances these have immortalized them. But they have transmitted to us the heritage of this organization which it is ours to enjoy, and which should be preserved immaculate and continued true to its original purpose.

Eight sessions of the International Ophthalmological Congress have now been held, as follows: the first at Brussels, September 13–16, 1857, under the presidency of Dr. L. S. Fallot, of Brussels; the second at Paris, September 30 and October 1–3, 1862, Dr. J. F. Vleminckx, of Brussels, president, and Dr. Jules Sichel, of Paris, perpetual honorary president; the third at Paris, August 12–14, 1867, Dr. Albrecht von Graefe, of Berlin, president; the fourth at London, August 1–3, 1872, Dr. F. C. Donders, of Utrecht, president; the fifth at New York, September 12–14, 1876, Dr. E. Williams, of Cincinnati, president; the sixth at Milan, September 1–4, 1880, Dr. Quaglino, of Milan, president; the seventh at Heidelberg, August 8–11, 1888, Dr. F. C. Donders, of Utrecht, president, for the second time; the eighth at Edinburgh, August 7–11, 1894, Dr. D. Argyll-Robertson, president. Each of these sessions has borne rich fruits, each has had a distinguished membership, each has been promotive of scientific research, and each has fostered that spirit of fraternity and good-will which extends far beyond town, city or country, and becomes truly international.

THE EIGHTH CONGRESS.

The eighth session of the congress, so recently held in Edinburgh, in whose proceedings it was my happy privilege to join, was common with the others in having a membership which included many of the most eminent ophthalmologists of the world and in presenting a program which included papers of the highest scientific rank. To be sure, none of the former presidents were there; indeed, they all live only in their works and the memories of those who knew them. Very many others who were active in other sessions and contributed so generously and acceptably had either been laid to rest or were stricken with disease and were giving their lives back to the Great Source of all life, as in the case of the revered and immortal Helmholtz, or were called elsewhere by various attractions and duties, and they were not there. They were missed. Professor Zehender, of Munich, was the only one present who was a member of the first congress, in 1857. The themes and questions which so aroused the enthusiasm, and so stimulated the thought and discussion of other meetings, were not introduced at this time. But there were new faces to take the place of old ones, and the subjects here discussed were as alive as those that made up the old programs, and its work was entered into with that zeal, and was possessed of that high scientific character which marked former meetings. While, therefore, the older generation of ophthalmologists is passing away, the newer is taking its place, and thus the vitality of the congress is preserved and its purposes continued.

Among the members of former congresses we saw the venerable Prof. Carlo Reymond, of Turin, and Dr. Zehender, of Munich. Then there were also Chibret, of Clermont-Ferrand; Deutschmann, of Hamburg; Holtenhoff, of Geneva; Hansen-Grut, of Copenhagen; Hirschberg, of Berlin; Th. Leber, of Heidelberg; Meyer, Landolt and Bull, of Paris; Priestly Smith, of Birmingham; Pfluger, of Berne; Sattler, of Leipzig; Schmidt-Rimpler, of Gottingen; Snellen, of Utrecht; Power, McHardy and A. Critchett, of London; Swanzy, of Dublin; and from our own country, Noyes, Knapp and Roosa, of New York, and Green, of St. Louis. To this list of men, so well known throughout the world, I might add others, but I mention these to show how truly the old is brought forward to the new membership, the one to be assimilated with the other to preserve the organic individuality of the past.

The membership of the Edinburg congress compared in numbers most favorably with that of other sessions. The number of listed members was 290, but the actual attendance, as shown by the registry on the afternoon of the last day, was about 170. The number listed from America, including Canada, was 69, of whom 32 from the United States and 5 from Canada were in actual attendance.

This session was held in Edinburgh, pursuant to an invitation extended by Dr. Argyll-Robertson, of that city, at the Heidelberg meeting, in 1880. The committee of organization was formed principally of a few Edinburgh physicians, with Dr. Argyll-Robertson, president, and Dr. George A. Berry general-secretary. This committee received the coöperation of the medical profession and many of the citizens of Edinburgh in perfecting plans for receiving and entertaining the gentlemen who might attend the congress, together with the ladies who might accompany them.

The renowned University of Edinburgh, with its medical school made equally famous by the long array of distinguished men who have honored it from its inception, in 1724, to the present time, opened wide its doors and tendered its memorable halls to the occupancy of the congress.

Fortified by such local support, the committee proceeded, early in 1894, to issue the call for the meeting, and invited contributions from men in all parts of the civilized world. From 9 to 1 each day, August 7–11, the congress was to devote itself to scientific work, and the balance of each day was to be given to entertainments of various kinds. In the latter, the committee commanded numerous resources and offered a "bill of fare" such as could not be provided by any other city but Edinburgh, possessed as it is of unexcelled beauties; interesting as it is in the annals of history; rich as it is in the numerous monuments, standing or crumbling, marking the struggles, the defeats, the despairs, the victories and the securities of a hardy and courageous people; distinguished as as it is as a great seat of learning and as a center of medical thought and culture, made lustrous by the Monros, Cullen, Gregory, Syme, Goodsir, Bennett, Christison, Simpson, Gairdner and many others; and surrounded as it is by a great number of places and objects that poets and historians have made famous by song and pen. With attractions like these, who could decline the invitation to "come?"

(*To be continued.*)

